# An optimized approach for increasing lesion size in temperature‐controled setting using a catheter with a surface thermocouple and efficient irrigation

**DOI:** 10.1002/joa3.13040

**Published:** 2024-04-22

**Authors:** Masateru Takigawa, Junji Yamaguchi, Masahiko Goya, Hidehiro Iwakawa, Tasuku Yamamoto, Miki Amemiya, Takashi Ikenouchi, Miho Negishi, Iwanari Kawamura, Kentaro Goto, Takatoshi Shigeta, Takuro Nishimura, Tomomasa Takamiya, Susumu Tao, Katsuhiro Ohuchi, Sayaka Suzuki, Shinsuke Miyazaki, Tetsuo Sasano

**Affiliations:** ^1^ Department of Cardiovascular Medicine Tokyo Medical and Dental University Hospital Tokyo Japan; ^2^ Division of Advanced Arrhythmia Research Tokyo Medical and Dental University Hospital Tokyo Japan; ^3^ Department of Cardiovascular Medicine Akita University Graduate School of Medicine Akita Japan; ^4^ Center for Experimental Animals Tokyo Medical and Dental University Tokyo Japan; ^5^ Japan Small Animal Medical Center Saitama Japan

**Keywords:** catheter ablation, half‐normal saline, high‐power short‐duration, normal saline, radiofrequency

## Abstract

**Background:**

We explore an optimized approach for increasing lesion size using a novel ablation catheter with a surface thermocouple and efficient irrigation in a temperature‐control setting.

**Methods:**

We conducted radiofrequency applications at various power levels (35 W, 40 W, and 45 W), contact forces (CFs, 10 g/20 g), and durations (60 s/120 s/180 s) in perpendicular/parallel catheter orientations, with normal saline irrigation (NS‐irrigation) and Half NS‐irrigation (HNS‐irrigation) in an ex‐vivo model (Step 1). In addition, we performed applications (35 W/40 W/45 W for 60 s/120 s/180 s in NS‐irrigation and 35 W/40 W for 60 s/120 s/180 s in HNS‐irrigation) in four swine (Step 2), evaluating lesion characteristics and the occurrence of steam pops.

**Results:**

In Step 1, out of 288 lesions, we observed 47 (16.3%) steam pops, with 13 in NS‐irrigation and 34 in HNS‐irrigation (*p* = .001). Although steam pops were mostly observed with the most aggressive setting (45 W/180 s, 54%) with NS‐irrigation, they happened in less aggressive settings with HNS irrigation. Lesion size significantly increased with longer‐duration ablation but not with HNS‐irrigation. The optimal %impedance‐drop cutoff to predict steam pops was 20% with a negative‐predictive‐value (NPV) = 95.1% including NS‐ and HNS‐irrigation groups, and 22% with an NPV = 96.1% in NS‐irrigation group. In Step 2, similar to the ex‐vivo model, lesion size significantly increased with longer‐duration ablation but not with HNS‐irrigation. Steam pops were absent with NS‐irrigation (0/35) even with the largest %impedance‐drop reaching 31% at 45 W/180 s. All steam pops were observed with HNS‐irrigation (6/21, 29%). The optimal %impedance‐drop cutoff predicting steam pops was 24% with an NPV = 96.3% including both NS‐ and HNS‐irrigation groups.

**Conclusions:**

Rather than using HNS‐irrigation, very long‐duration of radiofrequency applications up to 45 W/180 s may be recommended to safely and effectively increase lesion dimensions using this catheter with NS‐irrigation.

## Graphical abstract

In both ex‐vivo and in‐vivo studies, the radiofrequency application in a temperature‐controlled setting using a catheter equipped with a surface thermocouple and efficient irrigation demonstrates an increase in lesion size as the ablation duration extends up to 180 s. However, the utilization of half‐normal saline irrigation may not lead to a proportional increase in lesion size, but with more frequent steam‐pops.

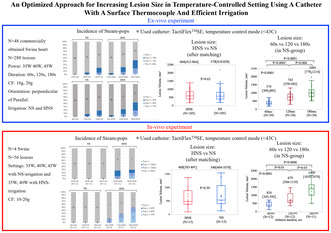

## 1 INTRODUCTION

Radiofrequency (RF) catheter ablation stands as an established treatment for cardiac arrhythmias. Ensuring optimal lesion formation and avoiding excessive heating is imperative for effective ablation.^1^ However, lesions generated through standard RF‐ablation techniques may have limited depth, posing a challenge to the efficacy of ablation in regions situated deep within the myocardium.^2^ These challenging areas include the interventricular septum, left ventricular summit, midmyocardial sites, and papillary muscles.^3^ Various strategies, such as coronary arterial or venous ethanol injection, RF ablation via a needle catheter, bipolar ablation, surgical subendocardial resection, and cardiac radiation are optional.^4^, ^5^, ^6^ However, the adoption of these techniques necessitates specialized expertize and equipment that may not be widely available, potentially limiting their feasibility and efficacy. On the other hand, prolonged high‐power RF ablation and HNS‐irrigation are relatively more convenient strategies to enlarge the lesion size. Several reports have demonstrated the impact of HNS‐irrigation on enlarging the lesion size in both ex‐vivo and in‐vivo experiments.^4^, ^7^, ^8^, ^9^ However, limited catheter platforms are used with power‐control settings in most of these studies, and there is another study with conflicting results.^9^ One clinical study showed the usefulness of using HNS irrigation in ventricular arrhythmias refractory to the conventional ablation strategy, but the incidence of steam pops was reported to be possibly associated with the catheter platform and the manner of irrigation.^10^

Multiple factors, including power, duration, catheter‐tissue contact force (CF), electrode diameter, circuit impedance, irrigation flow, tissue thickness, and myocardial blood flow, have been reported to be associated with lesion characteristics and steam pops^11^, ^12^, ^13^, ^14^ Therefore, the catheter platform, including the manner of irrigation and power regulation, may play a role. The TactiFlex™ SE (Abbott, St. Paul, MN, USA) is an innovative irrigation catheter equipped with a laser‐cut flexible tip and a CF sensor. With the catheter incorporating a single thermocouple positioned in extreme proximity to the tip for precise temperature monitoring, effective convective cooling is achieved through efficient irrigation flow, irrespective of the tissue‐catheter interface angle. This feature enables a safer and more continuous application of RF with temperature control settings. Our objective was to elucidate a strategy for safely creating deep lesions using this novel technology.

## 2 METHODS

### 2.1 RF‐applications

**FIGURE 1:**
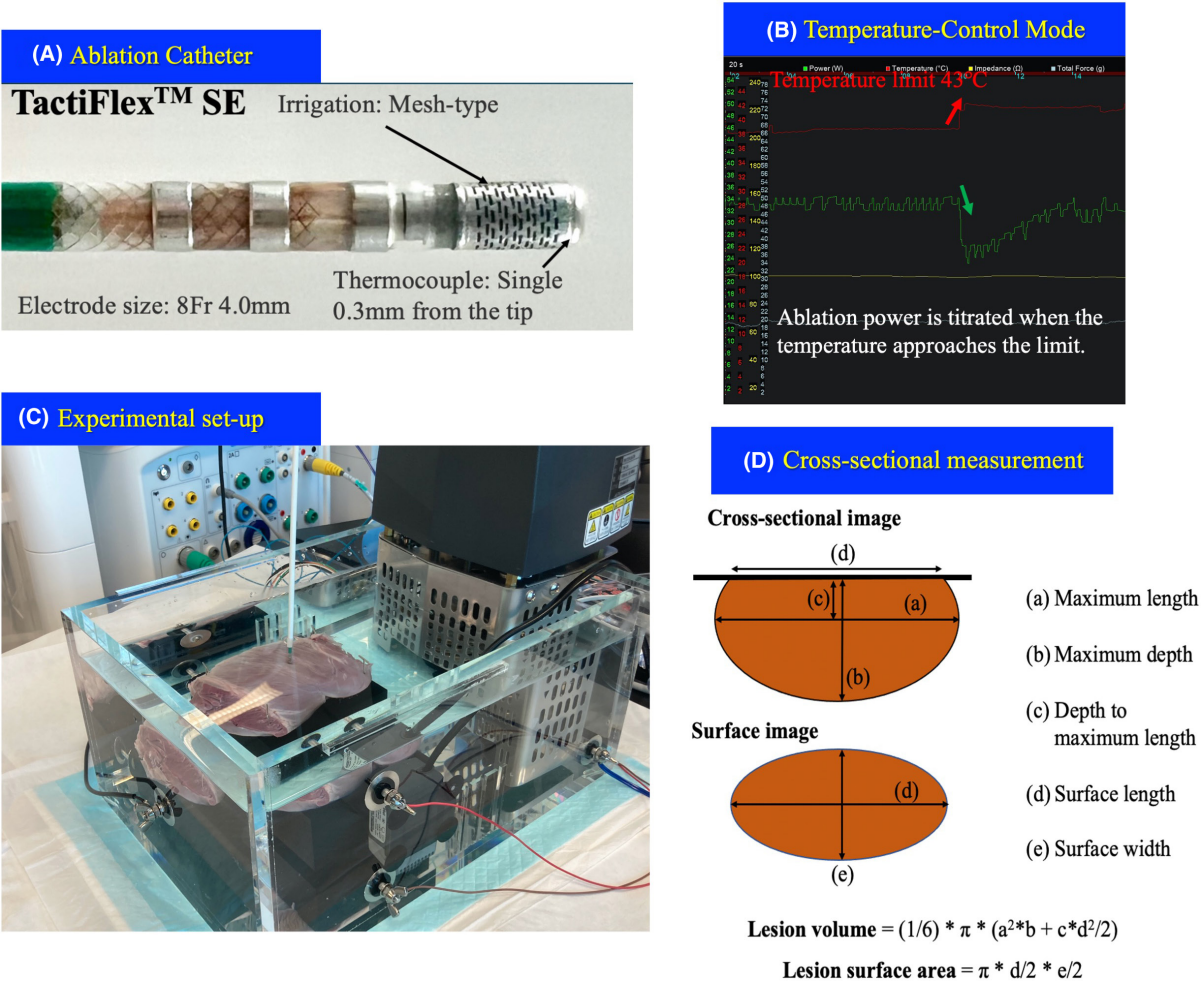
Experimental set‐up. (A) Catheter design of the TactiFlex™ SE ablation catheter with a contact sensor, installing a laser‐cut 4‐mm flexible 8Fr tip, which is irrigated from the proximal to distal end through the laser‐cut kerfs. A single thermocouple embedded within 0.3 mm from the tip for temperature monitoring, enabling ablation with temperature control mode. (B) Temperature control setting: Power output from the generator is automatically modulated to maintain the temperature measured from the catheter at or below the user‐set value. (C) Ex‐vivo experimental model. (D) Scheme of the surface and cross‐sectioned lesion.

A calibrated roller pump (CoolPoint, Abbott Medical) was connected to the catheter, delivering a saline solution at a rate of 13 mL/min during RF delivery. The TactiFlex™ SE contact‐sensor ablation catheter, featuring a laser‐cut 4‐mm flexible 8Fr tip with a surface thermocouple (0.3 mm from the tip), irrigated from the proximal to distal end through laser‐cut kerfs and from four holes on the distal end of the tip, was employed (Figure 1A). An Ampere RF generator (St. Jude Medical) was connected to administer 550 kHz unmodulated sine‐wave RF energy pulses in a temperature‐controlled mode with a target temperature of 43°C, meaning that power is titrated when the temperature approaches this level (Figure 1B). Various power settings, contact intensities, RF durations, and catheter orientations were employed throughout the experiment, as detailed in the following sections.

### 2.2 Ex‐vivo experiment with swine excised hearts

As part of the Step 1 experiment, a total of 48 commercially obtained swine hearts excised within <24 h were preserved in a fresh state and utilized for this ex‐vivo experimental model. A segment of the porcine left ventricular myocardium was positioned on the ground plate in a circulating saline bath containing 5.0 L saline at 37°C (Figure 1C). A flow pump was set at 5 L/min to simulate the cardiac output in the clinical setting, and the salinity was controlled to maintain an impedance level of 100 ± 5 Ω, measured by the catheter above the myocardial slab, simulating values based on clinical experience.

For the creation of various lesion sizes, myocardial lesions were generated at distinct sites with CF set at 10 and 20 g, and target power settings set at 35, 40, and 45 W. At each CF and power setting, RF delivery was administered for 60, 120, and 180 s unless steam pops occurred. The catheter was positioned both perpendicular and parallel to the tissue, with different irrigations, including normal saline (NS) and HNS. A total of four lesions were created for each combination of settings, resulting in 288 lesions in total.

### 2.3 In‐vivo experiment with swine

The protocol for the in‐vivo experiment, serving as Step 2, received approval from the Institutional Animal Care and Use Committees of Tokyo Medical and Dental University (A2022‐179A). Four swine (2 females, aged 3–4 months; weight 50–65 kg) were sedated with an intramuscular injection of ketamine hydrochloride (10 mg/kg) and xylazine (2 mg/kg). Following inhalation of isoflurane, each swine was intubated, and anesthesia was maintained with 2%–3% isoflurane. Swine were ventilated using a respirator (Carestation/Carescape, GE Healthcare, Chicago, IL), employing room air supplemented with oxygen. An intravenous sheath was inserted into the internal jugular vein for drug and fluid infusion. Intravenous amiodarone was administered to prevent ventricular arrhythmias. Arterial blood gases were periodically monitored, and ventilator parameters were adjusted to maintain blood gases within physiological ranges. A decapolar catheter was positioned in the coronary sinus (CS) through the internal jugular vein. Right and left ventricular mapping was conducted using the Advisor™ HD Grid Mapping Catheter, Sensor Enabled™ (Abbott) during CS pacing, followed by radiofrequency applications using TactiFlex™ SE. Target power settings ranging from 35, 40 to 45 W, and target ablation durations of 60, 120, and 180 s, with CF set at 10–20 g, were scheduled with NS‐irrigation. The same protocol was implemented with HNS‐irrigation, but only with 35 and 40 W applications. Applications lasting 60 s (5 lesions including 35, 40, 45 W in NS‐irrigation and 35, 40 W in HNS‐irrigation, in each animal) were intended for the right ventricular septum, while those with longer durations (10 lesions in each animal) were intended for the left ventricle. In total, 60 lesions were planned. Specific anatomic locations were marked in each chamber, ensuring that lesions were at least 1.5 cm apart from each other to prevent overlap and facilitate clear delineation of each lesion during measurements.

### 2.4 Lesion measurement and comparison

Following RF delivery, animals were sacrificed and endomyocardial lesions were measured, as illustrated in Figure 1D. The myocardium was cross‐sectioned along the surface length at the level of each lesion, and the cross‐sectional area was measured, as depicted in Figure 1D. A digital caliper with a resolution of 0.1 mm was employed by a single observer, who was blinded to the lesion protocol. Surface area and lesion volume were then calculated using the following formulas^15^, ^16^, ^17^:

$$\text{Lesion volume}=\left(1/6\right)\times \pi \times \left(e^{2}\times d+c\times a^{2}/2\right)$$

Lesion surface area=π×a/2×b/2

When comparing lesion sizes between the HNS‐irrigation and NS‐irrigation groups, ablation settings that led to steam pops in one cohort were excluded from the other to mitigate bias arising from the number of steam‐pop lesions. For instance, if the second lesion with 40 W/180 s in the HNS‐irrigation group resulted in a steam‐pop, the second lesion with 40 W/180 s in the NS‐irrigation group was also excluded from the comparison, even if this setting did not cause a steam pop. This matching is crucial because if many steam pops were observed in RF applications with higher power and longer duration in the HNS‐irrigation group, the NS‐irrigation group might show larger lesions only due to this bias. Similarly, when comparing lesion sizes among the 60, 120, and 180 s ablation settings, those causing steam pops in one cohort were excluded from the other two. For example, if a steam pop was observed in the third lesion with 180 s/40 W/HNS‐irrigation, the third lesions with 120 s/40 W/HNS‐irrigation and 60 s/40 W/HNS‐irrigation were also excluded from the comparison, even if these settings did not cause steam pops in these two groups.

### 2.5 Measurement of impedance variation

Variations of ablation parameters during each RF application were exported from the Ensite‐X system, in which the impedance variations were automatically calculated based on the maximum (not the initial) and minimum impedances during each RF‐application.

### 2.6 Statistical analysis

The data are presented as mean ± SD or median [25th percentile–75th percentile]. Continuous variables were compared using the Wilcoxon test or the Kruskal–Wallis test. Categorical variables were compared using the Chi‐squared test or Fisher's exact test. Significant differences were further assessed using the Steel–Dwass test for pairwise multiple comparisons. A *p*‐value <.05 was considered statistically significant.

## 3 RESULTS

### 3.1 Incidence of steam pops and lesion metrics: HNS versus NS

**FIGURE 2:**
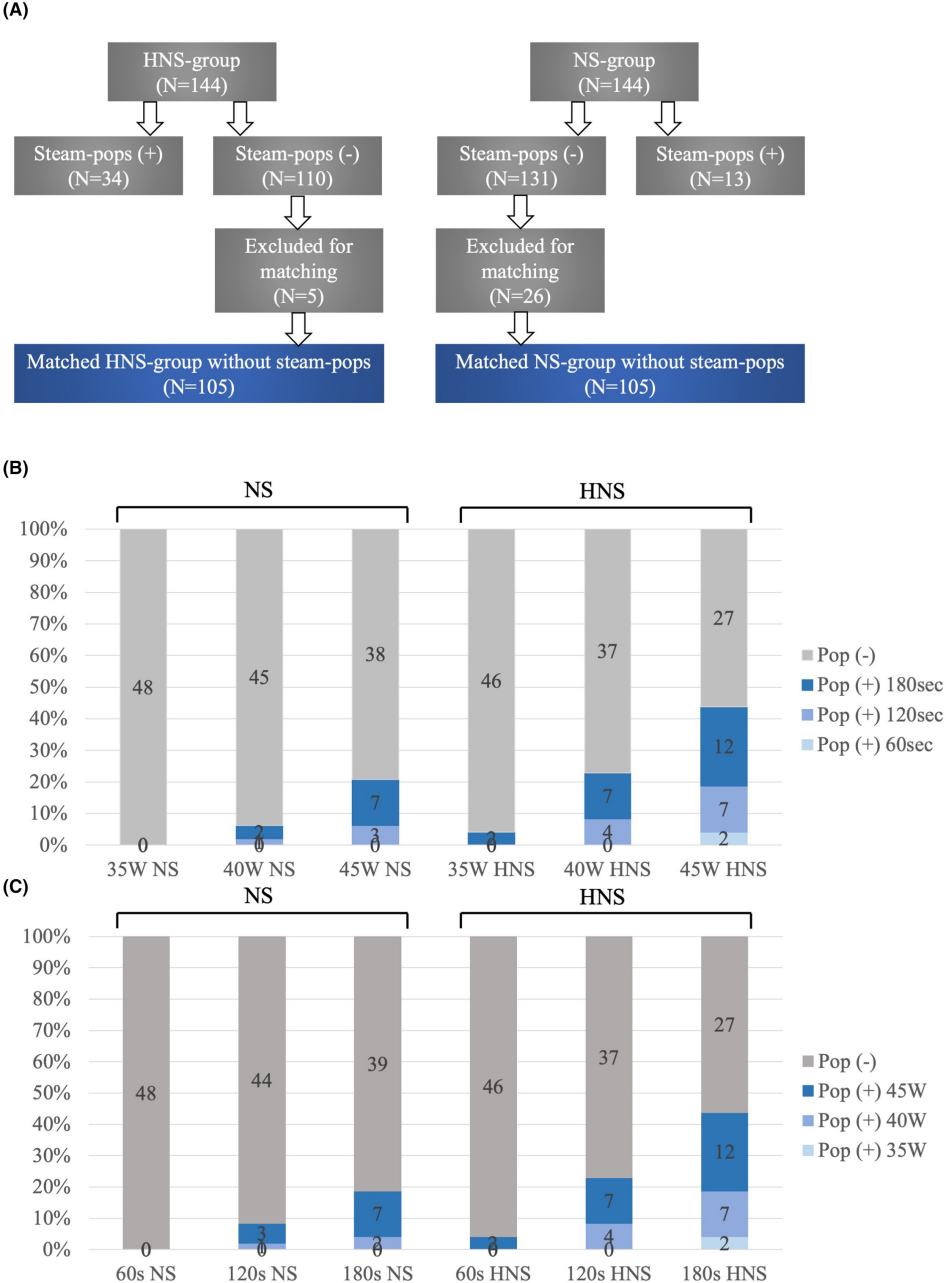
Incidence of steam pops in ex‐vivo condition. (A) Matching of the radiofrequency setting. (B) Ablation power versus steam pops. (C) Ablation duration versus steam pops.

**FIGURE 3:**
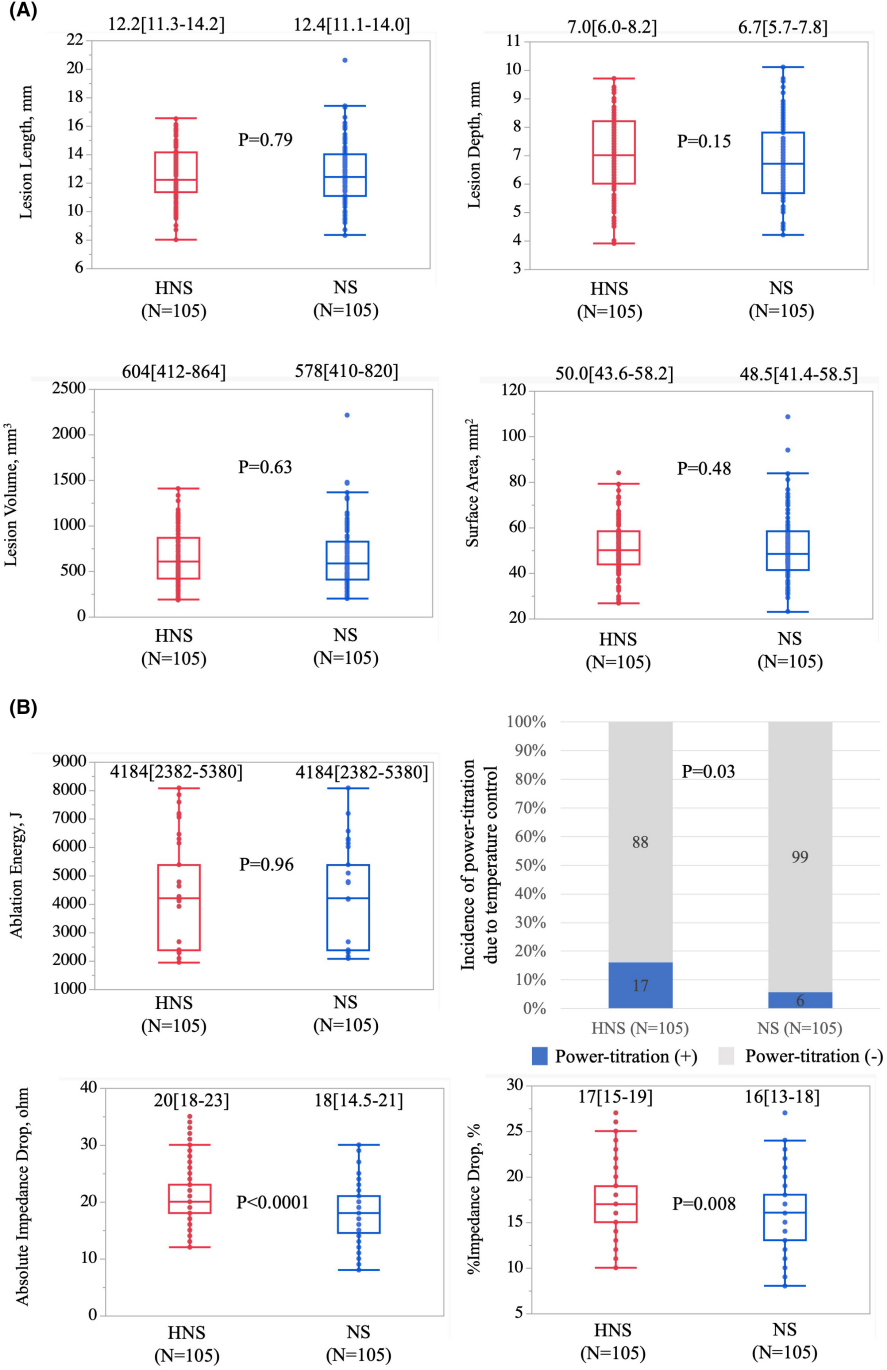
Comparison of lesion metrics and ablation parameters between HNS‐irrigation and NS‐irrigation in ex‐vivo model. (A) HNS‐irrigation versus NS‐irrigation: Lesion metrics. (B) HNS‐irrigation versus NS‐irrigation: Ablation parameters. HNS, half‐normal saline; NS, normal saline.

In total, 288 lesions were observed, including 47 (16.3%) steam pops, as illustrated in Figure 2A. Steam pops were more frequent when HNS was used for irrigation (13 [9.3%] in the NS group vs. 34 [23.6%] in HNS, *p* = .001). Although the incidence of steam pops tended to increase with higher targeted ablation power and duration, as depicted in Figure 2B,C, in the NS‐irrigation group, the most aggressive setting (45 W/180 s, *n* = 7/13, 54%) significantly contributed to steam pop occurrence, and the overall incidence of steam pops was generally low in other settings. After matching, 105 lesions produced with the same settings in each group were compared, showing no significant differences in length, depth, volume, and surface area of the lesions, as depicted in Figure 3A. In the HNS group, a significantly larger impedance drop was observed, and power titration due to the temperature control mode was more frequently observed, as shown in Figure 3B.

### 3.2 Incidence of steam pops and lesion metrics: 60 s versus 120 s versus 180 s

**FIGURE 4:**
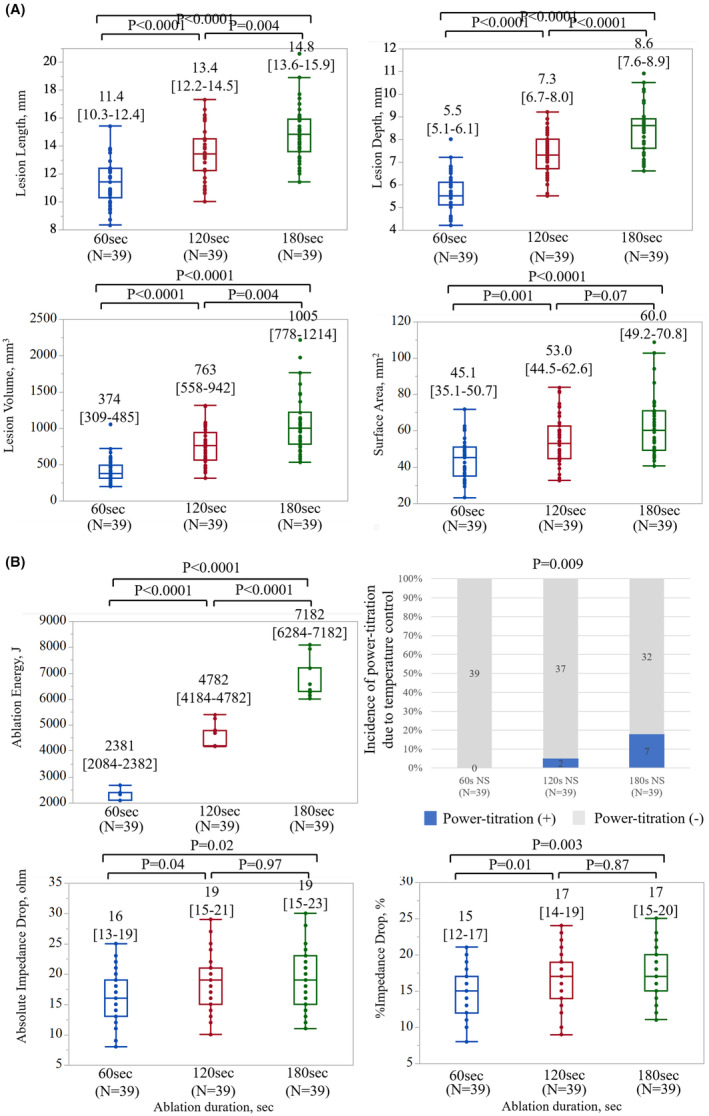
Comparison of lesion metrics and ablation parameters between three long RF‐applications with normal‐saline irrigation in ex‐vivo model. (A) Ablation duration versus Lesion metrics. (B) Ablation duration versus Ablation parameters. RF, radiofrequency.

As depicted in Figure 2B,C, the incidence of steam pops increased with the escalation of ablation duration and power in both the NS‐irrigation group and the HNS‐irrigation group. Tables 1 and 2 illustrates lesion metrics without steam pops depending on the targeted ablation duration and power. A comprehensive summary of the variation in ablation parameters during RF applications, both with and without steam pops, is provided in Table S1. Generally, lesion size increased proportionally with longer durations and higher power. However, for a precise analysis of the impact of long‐duration RF applications comparing lesion metrics among 60, 120, and 180 s, those with HNS‐irrigation were excluded to mitigate bias induced by the high incidence of steam pops. In addition, ablation settings causing steam pops in one cohort were excluded from the other two cohorts for matching. Ultimately, 39 lesions in each group were compared, revealing that the length, depth, volume, and surface area of the lesions significantly increased with prolonged ablation duration, as depicted in Figure 4A. Impedance drop was significantly smaller in 60 s but did not differ between 120 and 180 s, as shown in Figure 4B.

**TABLE 1:** Dimension of lesions without steam pops in each targeted power.

Lesion metrics	35 W‐NS (*n* = 48)	40 W‐NS (*n* = 45)	45 W‐NS (*n* = 38)	35 W‐HNS (*n* = 46)	40 W‐HNS (*n* = 37)	45 W‐HNS (*n* = 27)
Length, mm	12.3 ± 2.0	13.6 ± 2.3	13.7 ± 2.0	12.4 ± 1.8	12.9 ± 2.3	13.3 ± 2.2
Depth, mm	6.6 ± 1.5	7.3 ± 1.4	7.5 ± 1.5	7.0 ± 1.4	7.2 ± 1.3	7.2 ± 1.6
Volume, mm^3^	599 ± 301	799 ± 415	822 ± 378	630 ± 270	710 ± 327	733 ± 362
Area, mm^2^	47.7 ± 13.2	53.9 ± 15.0	60.0 ± 15.4	48.1 ± 11.1	53.1 ± 10.1	54.4 ± 12.8

Abbreviations: HNS, half‐normal saline; NS, normal saline.

**TABLE 2:** Dimension of lesions without steam pops in each targeted RF‐duration.

Lesion metrics	60 s NS (*n* = 48)	120 s NS (*n* = 44)	180 s NS (*n* = 39)	60 s HNS (*n* = 46)	120 s HNS (*n* = 37)	180 s HNS (*n* = 27)
Length, mm	11.4 ± 1.5	13.5 ± 1.7	14.8 ± 1.9	11.2 ± 1.4	13.5 ± 1.7	14.5 ± 1.4
Depth, mm	5.7 ± 0.8	7.4 ± 1.0	8.5 ± 1.1	5.9 ± 1.0	7.6 ± 0.8	8.5 ± 0.6
Volume, mm^3^	426 ± 155	772 ± 250	1067 ± 388	424 ± 152	773 ± 257	1000 ± 213
Area, mm^2^	44.1 ± 10.2	55.5 ± 13.5	62.4 ± 16.2	45.9 ± 10.0	52.7 ± 10.7	58.6 ± 10.4

Abbreviations: HNS, half‐normal saline; NS, normal saline; RF, radiofrequency.

### 3.3 Effect of catheter orientation

Out of 47/288 steam pops, 24/144 (16.7%) were observed in perpendicular placement and 13/144 (9.0%) were observed in parallel placement (*p* = .08). When the ablation settings causing steam pops were equally eliminated from both groups, and lesion metrics were compared between matched groups (*n* = 106 in each group), the surface lesion areas tended to be larger in parallel catheter placement (47.7 [41.5–53.4] mm in perpendicular vs. 49.7 [43.8–60.2] mm in parallel, *p* = .06), but the depth (6.9 [6–8] mm in perpendicular vs. 6.9 [5.8–8.1] mm in parallel, *p* = .81) and total volume (606 [411–835] mm in perpendicular vs. 576 [410–846] mm in parallel, *p* = .71) of the lesion were similar between the perpendicular and parallel placements. This tendency remained in both the NS‐irrigation group and the HNS‐irrigation group.

### 3.4 Confirmation in in‐vivo experiment

**FIGURE 5:**
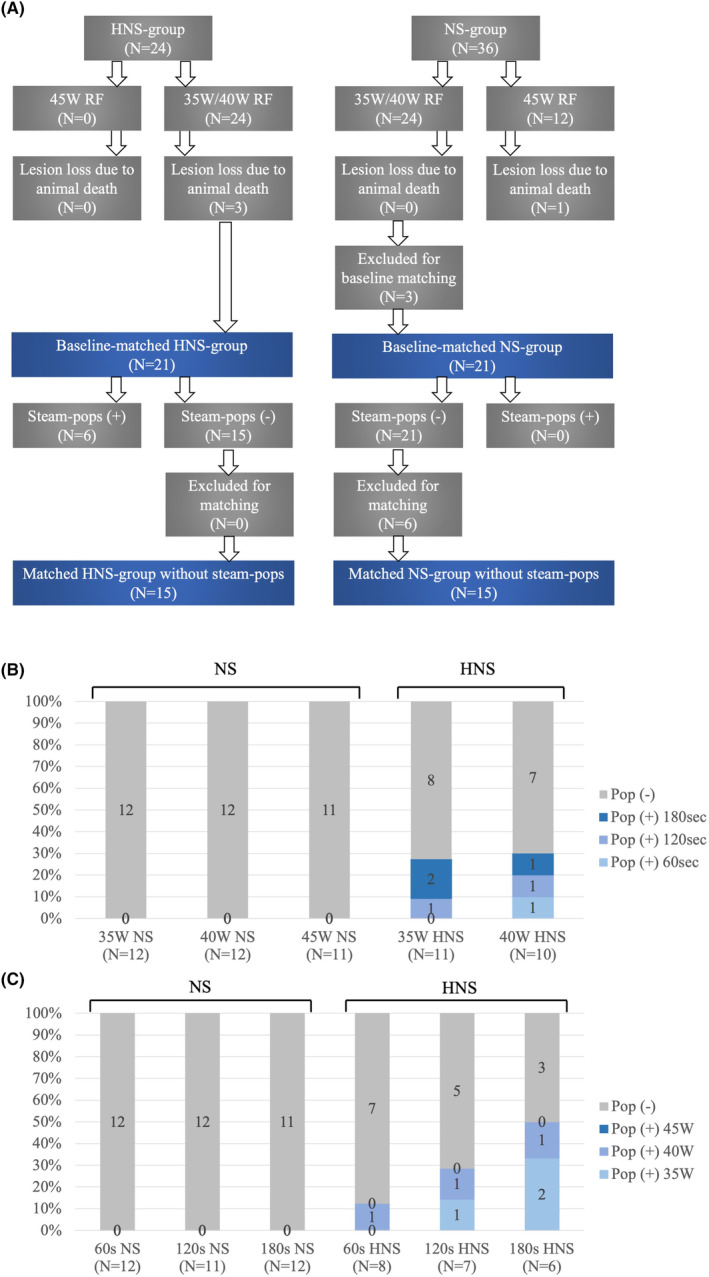
Incidence of steam pops in in‐vivo condition. (A) Matching of radiofrequency setting. (B) Ablation power versus steam pops. (C) Ablation duration versus steam pops.

While Swine 1, 3, and 4 completed the entire RF‐application protocol, certain settings (35 W/180 s, 40 W/120 s, 40 W/180 s with HNS, and 45 W/180 s with NS) were not performed in Swine 2 due to incomplete protocol execution resulting from the animal's demise. A total of 56 lesions were generated and all lesions were identified. As illustrated in Figure 5, six steam pops were exclusively observed in RF‐applications irrigated with HNS (0/35, 0% in NS vs. 6/21, 28.6% in HNS, *p* < .0001). Steam pops were noted in situations where the catheter was forced to be covered with the trabeculation or perpendicularly stacked at the base of the papillary muscle with a relatively larger contact force due to a small ventricular chamber.

**FIGURE 6:**
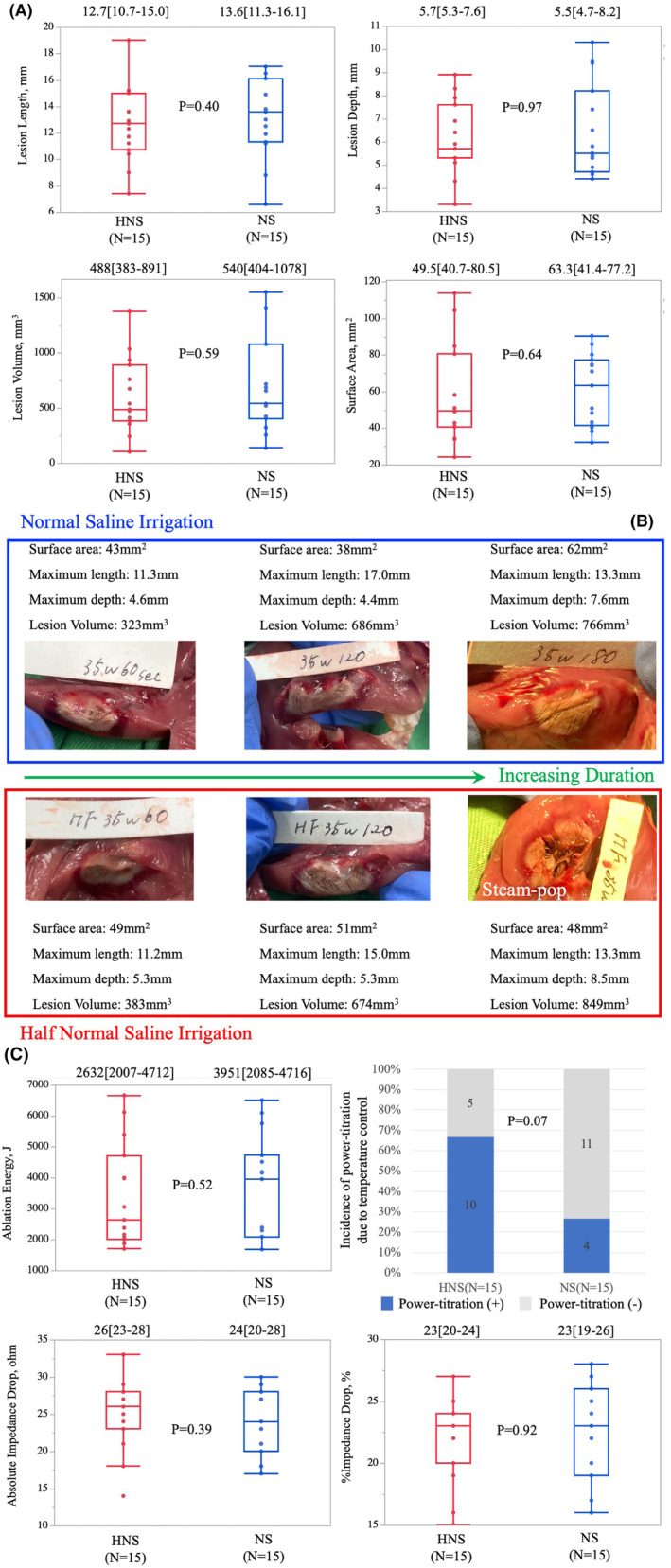
Comparison of lesion metrics and ablation parameters between HNS‐irrigation and NS‐irrigation in‐vivo model. (A) HNS‐irrigation versus NS‐irrigation: Lesion metrics. (B) HNS‐irrigation versus NS‐irrigation: demonstration of lesion metrics in different ablation settings. (C) HNS‐irrigation versus NS‐irrigation: Ablation parameters. HNS, half‐normal saline; NS, normal saline.

To prevent bias in the comparison of lesion metrics between the NS‐group and the HNS‐group, ablation settings causing steam pops and those not performed due to animal death in one cohort were excluded from the other cohort. The two groups were then matched, as depicted in Figure 5A. Ultimately, 15 lesions in the NS‐group and those in the HNS‐group were compared, revealing no significant differences in length, depth, volume, and surface area of the lesion between the two groups, as shown in Figure 6A,B, consistent with findings from the ex‐vivo study. Power titration due to temperature control was more frequently observed in HNS‐irrigation (10/15, 68%) than in NS‐irrigation (4/15, 27%) (*p* = .07) (Figure 6C).

**FIGURE 7:**
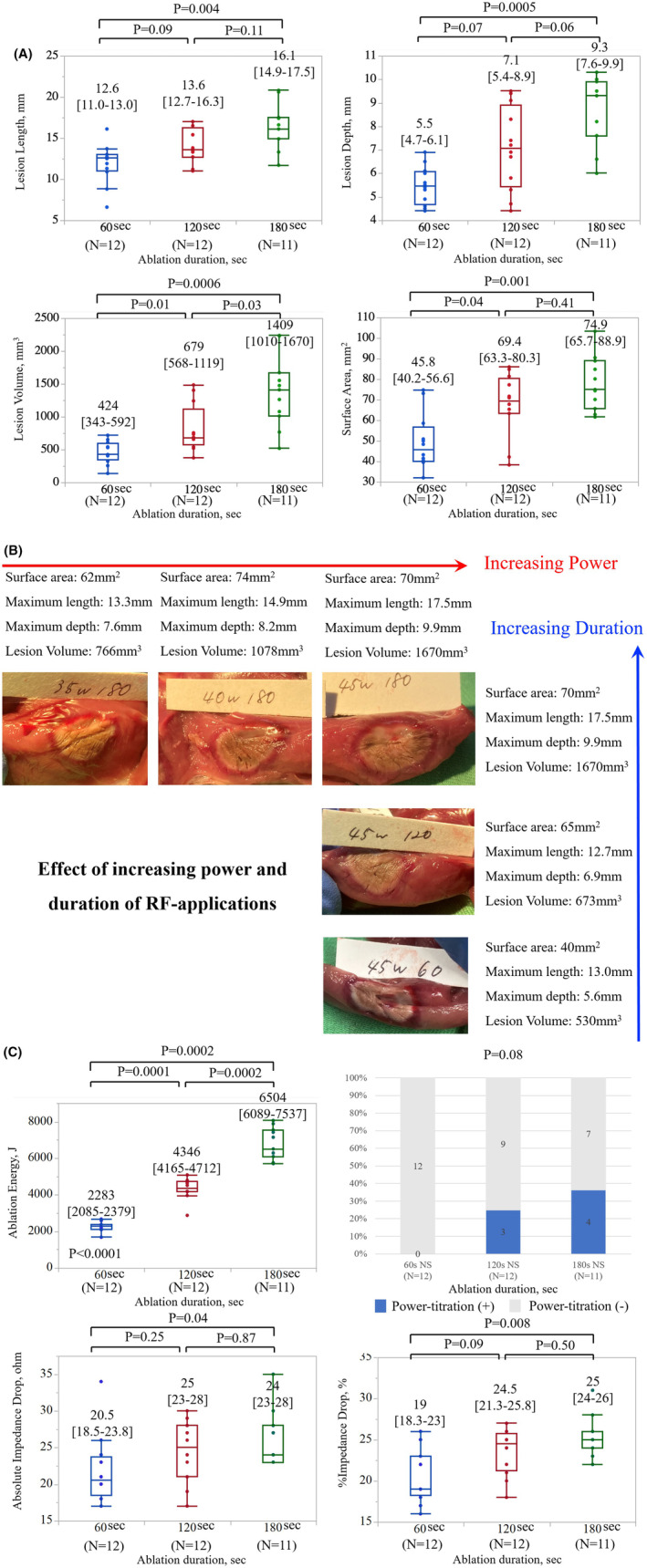
Comparison of lesion metrics and ablation parameters between three long RF‐applications in in‐vivo model. (A) Ablation duration versus Lesion metrics. (B) Demonstration of different lesion metrics in different ablation settings. (C) Ablation duration versus Ablation parameters. RF, radiofrequency.

The influence of prolonged ablation duration with this catheter is illustrated in Figure 7. Despite the omission of one application (45 W/180 s) due to the animal's demise and the limited total number of lesions, a notable trend of increasing lesion size was observed with an extended ablation duration (Figure 7A,B). While the impedance drop may be more substantial in the 120 s‐group compared to the 60 s‐group, no significant difference was observed between the 120 s group and the 180 s group (%impedance drop: 24.5 [21.3–25.8]% in the 120 s group vs. 25 [24–26]% in the 180 s group, *p* = .50).

### 3.5 Impedance variation versus steam pops

In the ex‐vivo experiment, among 288 lesions, 47 (16.4%) steam pops were observed, including 13 with NS‐irrigation and 34 with HNS‐irrigation. ROC curve analysis, encompassing both NS‐irrigation and HNS‐irrigation data, revealed an area under the curve (AUC) of 0.861 for a %Impedance‐drop in predicting steam pops (Figure S1A). The optimal cut‐off value for %Impedance‐drop was 20%, with sensitivity, specificity, positive predictive value (PPV), and negative predictive value (NPV) of 78.3%, 80.6%, 43.4%, and 95.1%, respectively. When the analysis was confined to the NS‐irrigation group, the AUC and optimal cut‐off value for %Impedance‐drop were 0.856% and 22%, respectively, with sensitivity, specificity, PPV, and NPV of 61.5%, 93.1%, 47.1%, and 96.1%, respectively (Figure S1B).

Figure S1C presents a comparison of impedance variation during RF‐applications between lesions with steam pops (*n* = 6) and those without (*n* = 50), incorporating both NS‐irrigation and HNS‐irrigation, in the in‐vivo experiment. The AUC and the optimal cut‐off value of %Impedance‐drop were 0.677% and 24%, respectively, with sensitivity, specificity, PPV, and NPV of 83.0%, 52.0%, 17.2%, and 96.3%. However, all six steam pops were observed with HNS‐irrigation. No steam pops occurred, even with 45 W/180 s RF‐applications in the NS‐irrigation group, despite the largest %impedance‐drop reaching 31%, meaning that the cut‐off value of %Impedance‐drop = 20%, derived from the ex‐vivo analysis, may remain a suitable threshold with a sufficient safety margin in an in‐vivo setting using NS‐irrigation with this catheter.

## 4 DISCUSSION

In this study, we explored a strategy to safely enhance the lesion size in temperature control mode using TactiFlex™ catheter, a CF‐sensing irrigation catheter with a surface thermocouple on the top, and a flexible laser‐cut tip with unique irrigation. Our findings revealed the following:

1. Lesion size demonstrated a proportional increase with RF duration of up to 180 s across power settings of 35, 40, and 45 W.
2. HNS‐irrigation did not consistently lead to a general increase in lesion size, as demonstrated in both ex‐vivo and in‐vivo studies.
3. Although longer‐duration radiofrequency applications suggested a potential increase in the risk of steam pops, as well as with HNS‐irrigation in the ex‐vivo experiment, this risk was limited in the in‐vivo environment, particularly with NS‐irrigation.
4. Although impedance generally decreased with a longer RF duration, the variation of impedance became limited with an RF‐duration >120 s regardless of the enlargement in lesion size.
5. In the in‐vivo setting, no steam pops occurred, even with 45 W/180 s RF‐applications in the NS‐irrigation group, despite the largest % impedance drop reaching 31%. A %impedance drop of 20% with both NS‐ and HNS‐irrigations and 22% with NS‐irrigation, derived from the ex‐vivo setting, maybe emerge as a reliable marker for predicting the incidence of steam pops with a sufficient safety margin in the in‐vivo setting.

Our study provides valuable insights into the factors influencing lesion formation and the associated risks, shedding light on potential strategies to optimize ablation outcomes.

### 4.1 How to safely create deep lesions

Although radiofrequency applications are reported to be more safely achieved using a catheter featuring an innovative irrigation manner with a surface thermocouple,^18^, ^19^ the optimized strategy to increase lesion size without raising the risk of steam pops to eliminate arrhythmogenic regions deep within the myocardium has still posed a significant challenge.^2^, ^3^ While longer RF applications were traditionally thought to enlarge lesion size, existing evidence primarily relied on power‐controled modes with standard irrigation catheters, often capping application duration at 60 s.^8^, ^9^, ^10^, ^20^, ^21^ Systematic experiments addressing whether very‐long RF applications can significantly increase lesion size without elevating the risk of steam pops have been lacking. In this study, we unequivocally demonstrated that RF applications lasting more than 60 s (e.g., 60, 120, and 180 s) may proportionally enhance lesion size in moderate‐ to high‐power settings (35, 40, and 45 W) with temperature control mode, utilizing the TactiFlex™ catheter with its efficient irrigation owing to a flexible‐tip design with laser‐cut kerfs. A recent study utilizing ablation‐index guided ablation with the Thermo‐Cool SmartTouch Surround Flow catheter (3.5 mm) at 30 W power control demonstrated that lesion size increases with ablation duration, up to 5 min in ex‐vivo and 4 min in in‐vivo settings.^22^ On the contrary, another approach to achieving deeper lesions is through the use of HNS‐irrigation, which enhances lesion size by elevating current density. The concept of augmenting surrounding environmental impedance was initially described in 2015 by Nguyen et al., where RF‐ablation with HNS‐irrigation increased lesion size without a concurrent increase in steam pops.^7^ However, evidence on this matter remains conflicting. Bennett et al. compared lesion size and the frequency of steam pops in 14 ovines using a 3.5‐mm irrigation catheter (30 W, 60 s, 10 g contact force, and 8 mL/min irrigation flow rate) between NS‐irrigation, HNS‐irrigation, and dextrose 5% in water (D5W)‐irrigation.^8^ They found that catheter ablation with HNS‐irrigation and D5W‐irrigation resulted in larger ablation lesions compared to NS‐irrigation but similar lesion dimensions between HNS‐irrigation and D5W‐irrigation. Furthermore, the incidence of steam pops was similar between HNS‐irrigation and NS‐irrigation, while D5W‐irrigation significantly increased steam pops. In a clinical study, the use of HNS‐irrigation demonstrated safety and effectiveness in 94 PVC/VTs refractory to ablation with NS‐irrigation, although steam pops were observed in 13%.^10^ Tschabrunn et al. compared lesion size and the incidence of steam pops between NS‐irrigation and HNS‐irrigation in 16 swine using a 3.5‐mm irrigation catheter (40 W with 10‐s ramp, 30‐s duration, 15 mL/min flow, and 8‐ to 14‐g target contact force). They found that HNS‐irrigation increased the incidence of steam pops but did not increase lesion size.^21^

Since lesion size and steam pops may be associated with the total current density and speed of the current flowing into the tissue, and irrigation for cooling the surface, the impact of HNS‐irrigation may differ according to the catheter platform and the volume and manner of irrigation, as discussed in a previous report.^10^, ^21^ Although, in the present study, HNS‐irrigation significantly increased the risk of steam pop, RF applications up to 180 s with a power range of 35–45 W especially with NS‐irrigation may augment lesion size with a limited risk of steam pops. With the TactiFlex™ catheter, long‐duration radiofrequency applications may be safer and more effective in increasing lesion dimensions and are recommended rather than using HNS‐irrigation.

### 4.2 Impedance variation for predicting lesion size

The proportional impact of longer RF‐application durations on lesion volume in the in‐vivo experiment did not achieve statistical significance but demonstrated a similar tendency to the ex‐vivo experiment. This effect was notably pronounced in the ex‐vivo experiment, where a sufficient number of lesions were attained for statistical significance. Intriguingly, impedance variations, such as absolute impedance drop and %impedance drop, did not proportionally increase. Particularly, there was no difference in impedance variation between 120 and 180 s applications in both ex‐vivo and in‐vivo experiments in the present study. As mentioned above, Bennet et al. compared lesion size in 14 ovine subjects using NS‐irrigation, HNS‐irrigation, and nonionic 5% dextrose (D5W)‐irrigation, showing that Impedance variation was largest in D5W‐irrigation, followed by HNS‐irrigation, and smallest in NS‐irrigation. Lesion size was smallest in NS‐irrigation, but no difference was observed between D5W‐irrigation and HNS‐irrigation.^8^ Younis et al. conducted both ex‐vivo and in‐vivo experiments to elucidate the reliability of ablation index‐guided ablation in ventricular myocardium using Thermo Cool Smart Touch Surround Flow catheter (30 W, 8 mL/min flow, and 15 g target contact force). Their data demonstrated that impedance variation reached a plateau before the lesion size reached a plateau.^22^ Although the lesion size results from the combination of resistive heating, convective heating, and convective cooling,^21^, ^23^, ^24^, ^25^ impedance variation may exhibit greater sensitivity to resistive heating effects compared to alterations occurring deep within the tissue, where convective heating has a more pronounced impact. The observed decrease in impedance with prolonged RF duration is closely linked to the temperature dynamics monitored at the catheter tip. This catheter design incorporates innovative irrigation techniques that effectively cool superficial tissue layers, mitigating the risk of steam pops. Meanwhile, deeper tissue regions undergo gradual heating through convective mechanisms, albeit not fully captured by the thermocouple at the catheter tip. While impedance variation might not serve as a very reliable predictor of lesion size in very‐long duration RF applications, further examination is required.

### 4.3 Impedance variation for predicting steam pops

On the other hand, impedance variation may be useful for predicting steam pops. A cut‐off %impedance drop >20% highly predicted the risk of steam pops with a high negative predictive value (NPV) of 95.1% in RF‐applications with both NS‐ and HNS‐irrigations in the ex‐vivo setting. Focusing on RF‐applications with NS‐irrigation, a cut‐off %impedance drop >22% also highly predicted the risk of steam pops with a high NPV of 96.1% in the ex‐vivo setting. In the in‐vivo setting, no steam pops occurred, even with 45 W/180 s RF‐applications in the NS‐irrigation group, despite the largest % impedance drop reaching 31%. Previously, we reported that the cut‐off value of 20% was optimal for predicting steam pops in ex‐vivo experiments with RF‐applications up to 60 s,^19^ and the clinical data showed that this value could be applied with a sufficient safety margin, primarily validated by RF‐applications with NS‐irrigation.^19^

Based on this evidence, we generally believe that a %impedance drop of 20% with both NS‐ and HNS‐irrigations and 22% with NS‐irrigation remains a reliable cutoff value to avoid steam pops, demonstrating a high negative predictive value (NPV), even with longer RF applications (>60 s). However, in some applications during the ex‐vivo experiment and those involving HNS irrigation in in‐vivo experiments, steam pops occurred even with a lower %impedance drop ≤20%. The occurrence of steam pops is not solely linked to the total delivered energy or current to the tissue but is also influenced by the speed of tissue heating.^26^, ^27^ Although ablation power is titrated in temperature control mode, the rapid increase in current density may lead to steam pops before the power titration due to the temperature control setting is activated. Therefore, it is crucial to emphasize that in conditions where a significant portion of the catheter tip is presumed to be covered with cardiac tissue^27^ such as trabeculation, in high initial impedance, or the use of HNS‐irrigation, RF applications should be performed cautiously. This is because a substantial portion of the electric current may rapidly flow into the tissue without dissipating to the blood pool, resulting in steam pops before reaching the %impedance of 20%.

### 4.4 Novelty of the present study and clinical implications

As demonstrated in previous clinical studies,^9^, ^10^, ^21^, ^28^ the safety and efficacy of HNS irrigation may vary depending on the catheter platform, including factors such as electrode size and material, location and the number of thermocouples, volume, and method of irrigation, and the ablation mode (e.g., power‐controlled vs. temperature control). It is conceivable that the use of HNS‐irrigation may be safer and more effective than increasing the power of duration with one catheter platform, while the opposite may be true for another catheter platform.

The present study specifically focused on the TactiFlex™ SE catheter, which features a relatively larger electrode tip (4 mm) with a surface thermocouple and a unique irrigation system, usually used with temperature‐control mode. Further, the effect of long‐RF applications up to 180 s and HNS‐irrigation on lesion creations was systematically examined. The findings suggest that, with this catheter, extending the duration and power of RF application may be recommended over using HNS‐irrigation to safely achieve larger and deeper lesions. The in‐vivo experimental model demonstrated that lesions could be enlarged, and no steam pops were observed even with 45 W/180 s applications. However, it is crucial to exercise caution when applying this maximum setting, as some steam pops were observed in the ex‐vivo setting. It is worth noting that the ex‐vivo environment may exhibit a higher frequency of steam pops due to the reduced convective cooling effect.

### 4.5 Limitations

First, these experiments were conducted using either an ex‐vivo model or an in‐vivo model with healthy swine ventricular myocardium. Therefore, the results obtained may not necessarily generalize to clinical human pathology. Conducting a human study with histopathology may pose feasibility challenges. Second, it is important to note that RF applications were predominantly performed in the left ventricle in the present study, and as a result, the findings may not universally apply to other cardiac chambers. However, in clinical practice, very long duration applications, such as 3 min, are commonly used for the left ventricle. Third, it should be noted that the findings of this study may be limited in their applicability to the TactiFlex™ SE catheter, potentially lacking generalizability. Nevertheless, it is crucial to recognize that the effects of prolonged application and HNS‐irrigation are likely to be influenced significantly by the specific characteristics of the catheter platform. Thus, discussion of these effects should be tailored to each catheter individually. Finally, while “matching” may introduce another bias focusing on lower power, shorter duration conditions by removing lesions with longer duration or higher power lesions, it may be required to compare the unbalanced incidence of steam pops between NS‐irrigation and HNS‐irrigation.

## 5 CONCLUSIONS

Longer RF‐applications may be recommended instead of using half‐normal saline irrigation to safely achieve deeper lesions using a catheter with a surface thermocouple and unique irrigation in temperature‐control mode.

## FUNDING INFORMATION

This work was partially supported by JSPS KAKENHI Grant Number JP20K17074 and 22K16068.

## CONFLICT OF INTEREST STATEMENT

Drs. Goto, Takigawa, and Miyazaki belong to the division which receives research endowments from Medtronic Japan, Boston Scientific, Japan Lifeline, APEX, and WIN international. Dr. Takigawa received a lecture fee from Abbott Medical Japan, LIS. Dr. Ohuchi received a joint research fund from Abbott Medical Japan, LIS. No other authors have conflict of interest to declare.

## ETHICS STATEMENT

The study protocol of the animal experiment received approval from the Institutional Animal Care and Use Committees of Tokyo Medical and Dental University (A2022‐179A).

## Supporting information


Figure S1.

Table S1.

## Data Availability

The data underlying this article will be shared on reasonable request to the corresponding author.
