# Supplementary figures and images for: An optimized approach for increasing lesion size in temperature‐controled setting using a catheter with a surface thermocouple and efficient irrigation
Source: J Arrhythm. 2024 Apr 22;40(3):536–51. doi: 10.1002/joa3.13040 (PMC11199823; doi:10.1002/joa3.13040)

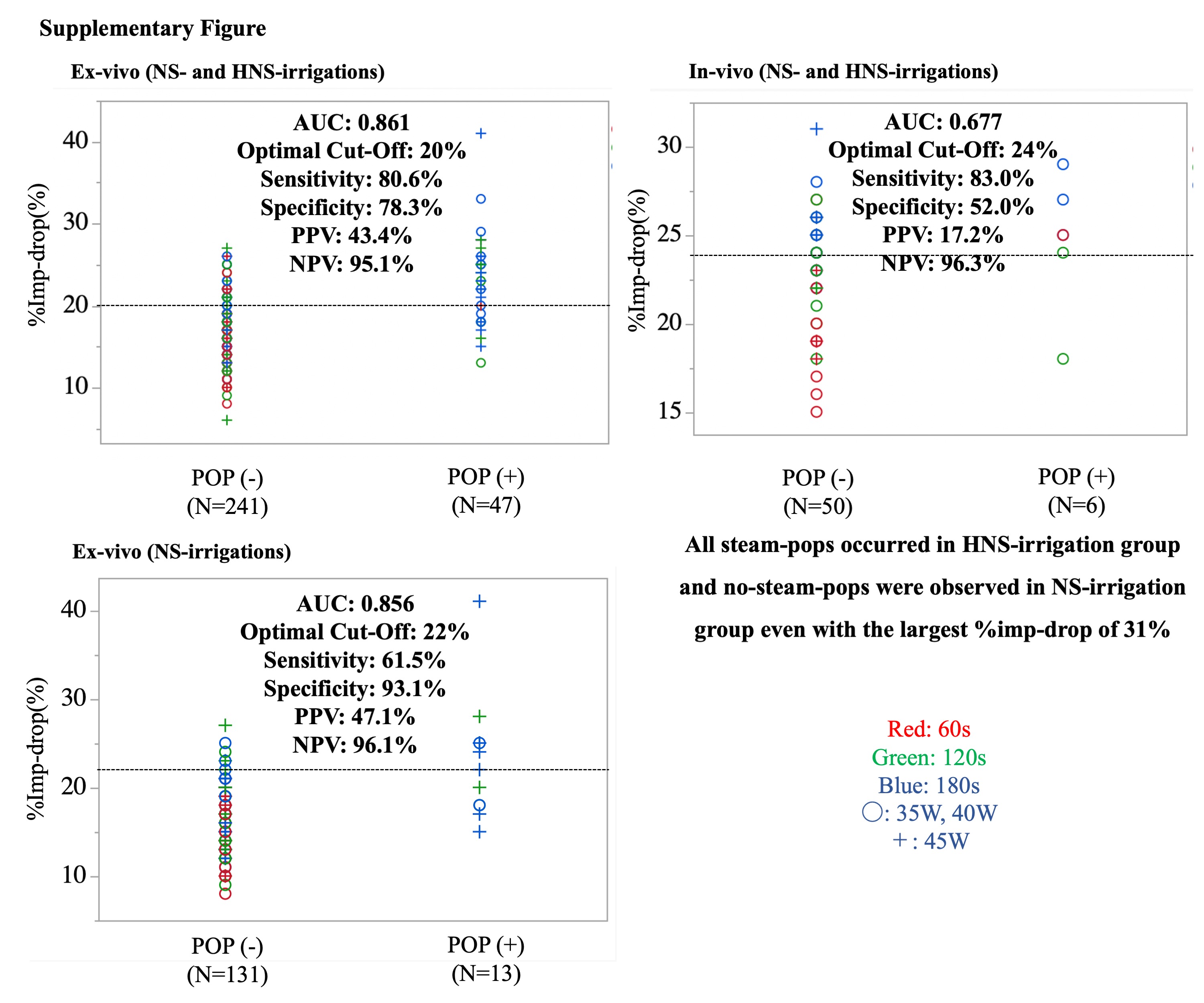

Supplement: Supplementary file 1 — Figure S1. [file JOA3-40-536-s001.jpg]
