# Supplementary material for: An optimized approach for increasing lesion size in temperature‐controled setting using a catheter with a surface thermocouple and efficient irrigation
Source: J Arrhythm. 2024 Apr 22;40(3):536–51. doi: 10.1002/joa3.13040 (PMC11199823; doi:10.1002/joa3.13040)
Supplement: Supplementary file 2 — Table S1. [file JOA3-40-536-s002.docx]

**Supplementary Table 1.**

**A. Variation in parameters with/without steam-pops in each targeted power**

| Excluding steam-pops | 35W-NS(n=48) | 40W-NS(n=45) | 45W-NS(n=38) | 35W-HNS(n=46) | 40W-HNS(n=37) | 45W-HNS(n=27) |
| --- | --- | --- | --- | --- | --- | --- |
| Delivered Energy, J | 4173+/-1722 | 4646+/-1973 | 4820+/-2101 | 4075+/-1708 | 4275+/-1907 | 4299+/-1866 |
| Average delivered power, W | 34.9+/-0.3 | 39.6+/-1.0 | 44.6+/-1.7 | 34.8+/-0.5 | 39.5+/-1.1 | 44.8+/-0.6 |
| Actual ablation duration, sec | 120+/-50 | 117+/-49 | 109+/-48 | 117+/-49 | 109+/-49 | 97+/-43 |
| Max temp, ℃ | 34.8+/-3.0 | 34.4+/-4.4 | 34.7+/-4.0 | 34.3+/-4.1 | 35.1+/-4.8 | 34.1+/-4.02 |
| Absolute impedance drop, ohm | 17.3+/-4.7 | 18.2+/-4.2 | 18.3+/-4.7 | 20.0+/-4.1 | 21.4+/-6.1 | 23.1+/-4.6 |
| %Impedance drop, % | 15.4+/-3.7 | 16.5+/-3.4 | 16.7+/-3.8 | 16.5+/-3.1 | 17.8+/-4.1 | 19.0+/-3.3 |
| Average CF, g | 15.2+/-4.9 | 14.8+/-5.0 | 15.2+/-5.1 | 15.1+/-4.9 | 14.7+/-4.8 | 13.6+/-4.9 |
| Power titration | 4(8.3%) | 6(13.3%) | 5(13.2%) | 6(13.0%) | 8(21.6%) | 2(7.4%) |
| Including steam-pops | 35W-NS(n=48) | 40W-NS(n=48) | 45W-NS(n=48) | 35W-HNS(n=48) | 40W-HNS(n=48) | 45W-HNS(n=48) |
| Delivered Energy, J | 4173+/-1722 | 4633+/-1923 | 4932;/-1999 | 4110+/-1684 | 4373+/-1732 | 4185+/-1761 |
| Average delivered power, W | 34.9+/-0.3 | 39.6+/-1.0 | 44.6+/-1.5 | 34.8+/-0.6 | 39.6+/-1.0 | 44.7+/-0.6 |
| Actual ablation duration, sec | 120+/-50 | 117+/-48 | 111+/-45 | 118+/-48 | 111+/-44 | 94+/-40 |
| Max temp, ℃ | 34.8+/-3.0 | 34.9+/-4.7 | 35.2+/-4.1 | 34.3+/-4.0 | 35.3+/-4.4 | 35.1+/-3.7 |
| Absolute impedance drop, ohm | 17.3+/-4.7 | 18.5+/-4.4 | 20.0+/-6.5 | 20.4+/-4.9 | 22.8+/-6.7 | 24.9+/-5.6 |
| %Impedance drop, % | 15.4+/-3.7 | 16.8+/-3.5 | 18.3+/-5.5 | 16.8+/-3.5 | 18.8+/-4.8 | 20.5+/-4.3 |
| Average CF, g | 15.2+/-4.9 | 15.1+/-5.1 | 15.1+/-5.0 | 15.0+/-4.9 | 14.8+/-4.7 | 14.8+/-4.8 |
| Power-titration | 4(8.3%) | 7(14.6%) | 6(12.5%) | 6(12.5%) | 10(20.8%) | 4(8.3%) |

CF, contact force; HNS, half-normal saline; NS, normal saline

**B. Variation in parameters with/without steam-pops in each targeted RF-duration**

| Excluding steam-pops | 60s-NS(n=48) | 120s-NS(n=44) | 180s-NS(n=39) | 60s-HNS(n=46) | 120s-HNS(n=37) | 180s-HNS(n=27) |
| --- | --- | --- | --- | --- | --- | --- |
| Delivered Energy, J | 2373+/-248 | 4724+/-479 | 6944+/-705 | 2361+/-247 | 4639+/-501 | 6719+/-565 |
| Average delivered power, W | 39.8+/-4.1 | 39.5+/-4.0 | 38.6+/-4.0 | 39.6+/-4.2 | 38.8+/-4.2 | 37.7+/-3.5 |
| Actual ablation duration, sec | 60 | 120 | 180 | 60 | 120 | 180 |
| Max temp, ℃ | 34.4+/-3.5 | 34.5+/-3.7 | 35.1+/-4.3 | 33.6+/-3.7 | 34.5+/-4.3 | 36.3+/-4.9 |
| Absolute impedance drop, ohm | 16.2+/-3.9 | 18.8+/-4.7 | 19.0+/-4.5 | 19.9+/-4.8 | 22.3+/-5.3 | 22.0+/-4.9 |
| %Impedance drop, % | 14.5+/-3.1 | 17.0+/-3.8 | 17.3+/-3.4 | 16.3+/-3.4 | 18.4+/-3.7 | 18.4+/-3.5 |
| Average CF, g | 15.2+/-4.9 | 15.2+/-5.0 | 14.7+/-5.1 | 15.0+/-4.9 | 14.6+/-4.9 | 14.0+/-4.7 |
| Power-titration | 3(6.3%) | 5(11.4%) | 7(25.9%) | 3(6.5%) | 6(16.2%) | 7(25.9%) |
| Including steam-pops | 60s-NS(n=48) | 120s-NS(n=48) | 180s-NS(n=48) | 60s-HNS(n=48) | 120s-HNS(n=48) | 180s-HNS(n=48) |
| Delivered Energy, J | 2373+/-248 | 4665+/-504 | 6702+/-1032 | 2359+/-245 | 4493+/-651 | 5816+/-1507 |
| Average delivered power, W | 39.8+/-4.1 | 39.8+/-4.0 | 39.5+/-4.2 | 39.8+/-4.2 | 39.7+/-4.3 | 39.7+/-4.0 |
| Actual ablation duration, sec | 60+/-2 | 118+/-8 | 171;/-24 | 60 | 114+/-15 | 150+/-42 |
| Max temp, ℃ | 34.4+/-3.5 | 34.9;/-4.0 | 35.6+/-4.4 | 33.7+/-3.6 | 35.1+/-4.1 | 36.0+/-4.1 |
| Absolute impedance drop, ohm | 16.2;/-3.9 | 19.4;/-5.2 | 20.3;/-6.0 | 20.3+/-5.2 | 23.1;/-6.1 | 24.7+/-5.9 |
| %Impedance drop, % | 14.5+/-3.1 | 17.4+/-4.2 | 18.5+/-5.0 | 16.7+/-3.7 | 19.0+/-4.5 | 20.5+/-4.3 |
| Average CF, g | 15.2+/-4.9 | 15.2+/-5.0 | 15.1+/-5.1 | 15.0+/-4.9 | 14.8+/-4.9 | 14.8+/-4.7 |
| Power-titration | 3(6.3%) | 5(10.4%) | 9(18.8%) | 3(6.3%) | 8(16.7%) | 9(18.8%) |
